# Endodontic Consequences of Early Stage of Medication‐Related Osteonecrosis of the Jaw: A Case Report

**DOI:** 10.1002/cre2.70168

**Published:** 2025-06-27

**Authors:** Vanessa Baaroun, Samantha Elbhar, Carole Rémond, Ines Guessoum, Juliette Rochefort, Geraldine Lescaille, Yves Boucher, Marjorie Zanini

**Affiliations:** ^1^ Department of Oral Surgery, School of Dentistry Université Paris Cité Paris France; ^2^ Pitie Salpêtrière Hospital Paris France; ^3^ Department of Endodontics, School of dentistry Université Paris Cité Paris France; ^4^ Liberal practice Asnières sur Seine France; ^5^ Department of Orofacial pain and Imagery, School of dentistry Université Paris Cité Paris France; ^6^ Laboratoire Santé Orale INSERM UMR 1333 Université Paris Cité France

**Keywords:** bisphosphonate‐associated osteonecrosis of the jaw/pathology, case reports, diagnostic errors, endodontics, pulpitis

## Abstract

**Objectives:**

Osteonecrosis of the jaws (MRONJ) is a frequent side effect of antiresorptive (AR) drugs used in oncology. MRONJ may have endodontic consequences, as reported in this clinical case.

**Material and Methods:**

A 64‐year‐old woman being treated with antiresorptive (AR) drugs targeting bone metastasis of a primitive breast cancer consulted at the dental service of Pitié‐Salpêtrière Hospital. She first experienced symptomatic apical periodontitis followed by symptomatic irreversible pulpitis, which were initially explained as resulting from occlusal trauma. Despite endodontic treatment, exacerbation of the symptomatology was noted. MRONJ was suspected, and the affected tooth was extracted.

**Results:**

The presence of necrotic bone during the surgery confirmed the diagnosis. Surgical treatment led to complete healing and total disappearance of clinical and radiological signs at 4 months.

**Conclusions:**

MRONJ can induce alterations in adjacent tooth vascularization and secondary pulpal disease. Early diagnosis is difficult because early‐stage MRONJ occurs without clear radiographic signs.

## Summary

- MRONJ is a side effect of antiresorptive (AR) drugs used in oncology. MRONJ may have endodontic consequences, as reported in this clinical case.
- Endodontists should be aware of the side effects of AR drugs and their possible consequences on dental pulp to avoid delayed diagnosis and invasive interventions.

## 1 Introduction

Antiresorptive (AR) drugs include bisphosphonates (BP) and more recently denosumab. BP molecules bind to bone mineral and are taken up by mature osteoclasts, eventually inducing osteoclast apoptosis. Denosumab is an immunoglobulin that blocks RANK ligand and prevents its binding to the RANK receptor involved in differentiation and function of osteoclasts (Baron et al. 2011). Both BP and denosumab inhibit osseous resorption, leading to their use in rheumatology for conditions such as Paget's disease, osteoporosis, and osteogenesis imperfecta, and in oncology for osseous metastasis and multiple myeloma.

While the therapeutic efficacy of both BP and denosumab is well established, their major side effect is the induction of MRONJ (Medication‐Related Osteonecrosis of the Jaw), either spontaneously or in conjunction with certain dental conditions (Ruggiero et al. 2014). MRONJ is clinically classified by the American Association of Oral and Maxillofacial Surgeons into four different stages (Table 1A) (Ruggiero et al. 2014). Stage 0 is defined by the “presence of nonspecific clinical findings and symptoms and clinical evidence of bone necrosis.” Thus, diagnosis may be difficult where there is a paucity of clinical signs and no bone exposure, and misdiagnosis as a periodontal or an endodontic disease may occur (Katz 2005). General local risk factors of MRONJ are listed in Table 1B. The presence or absence of these factors allows the practitioner to evaluate the specific risk of osteonecrosis for a patient (Nicolatou‐Galitis et al. 2019) (Table 1C).

**Table 1:** (A) Clinical classification of medication‐related osteonecrosis of the jaw (MRONJ), according to the AAOMS classification and 2014 update (2). (B) General and local risk factors of MRONJ. (C) Graduation of the risk of MRONJ.

A. Clinical classification of MRONJ	
Stage	Definition
0	Presence of nonspecific clinical findings and symptoms and clinical evidence of bone necrosis
1	Presence of exposed and necrotic bone in asymptomatic patients and no evidence of infection
2	Presence of exposed necrotic bone associated with infection (pain and erythema, with or without purulent drainage)
3	Presence of exposed necrotic bone, pain, infection and one of the following clinical manifestations: exposed and necrotic bone extending beyond the region of alveolar bone, resulting in pathologic fracture, extraoral fistula, oral antral/oral nasal communication or osteolysis extending to the inferior border of the mandible or the sinus floor

**B. Risk factors of MRONJ**	
**General Risk Factors**	
Indication of the AR medication prescribed.	
Duration of exposure and the cumulated administration dose.	
Concomitant pathology that can affect healing such as diabetes or rheumatoid arthritis.	
**Local Risk Factors**	
Poor oral conditions.	
Maxillary anatomy (especially tori).	
Traumatic – surgical – treatments (especially tooth extraction).	
Location (mandibular and/or molar region).	
Periodontal disease.	
**C. Graduation of the Risk of MRONJ**	
R0: patients eligible and not yet treated with AR medication or patients exposed to AR medications for less than 3 years, in the absence of other systemic or local risk factors	
RX: patients exposed to AR medication for more than 3 years or patients exposed to AR for less than 3 years but in the presence of other systemic or local risk factors.	

The aim of this article is to report a case of MRONJ initially presenting only clinical and radiological signs of periapical periodontitis, secondarily evolving toward pulpal disease. Endodontic consequences of AR medications are discussed.

## 2 Case Description and Results

This case report has been written according to CARE guidelines (Gagnier et al. 2013).

### 2.1 First Visit: Emergency Department

A 64‐year‐old woman consulted the Dental Emergency Unit (DEU) of Pitié Salpêtrière Hospital because of left mandibular pain when biting.

*Medical history:* The patient reported Parkinson's disease since 2003 and a primitive breast cancer (adenocarcinoma; HER2) diagnosed in 2009, treated by surgery combined with local radiotherapy and ongoing chemotherapy; a cancer recurrence associated with osseous metastasis in 2019 which led to chemotherapy and AR drugs for 6 months including one intravenous (IV) administration of alendronate (BP; Zometa 4 mg) followed by 5 IV administrations of denosumab (Xgeva; cumulative dose 600 mg). 4 weeks before the DEU visit, the oncologist advised withdrawal of AR medication to lower the risk of MRONJ.

*Dental history:* The patient self‐reported night bruxism. The patient had a Kennedy Class III posterior partial edentulism not compensated by a prosthetic device.

**Figure 1:**
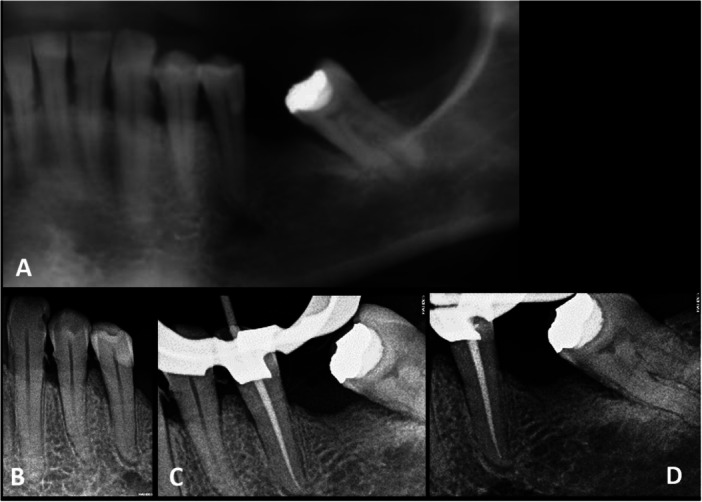
Initial radiological results of the lower left second premolar. (A) Panoramic X‐ray focused on the lower left second premolar, indicating a periapical index score of 2 for this tooth, presenting clinical signs of acute periapical periodontitis and normal response to pulpal tests. (B) Preoperative X‐ray showing a PAI score of 2. (C) Per operative X‐ray with gutta‐percha master cone. (D) Postoperative X‐ray.

*Clinical findings:* The pain was localized on Tooth#35 (VAS 5/10) and reproduced by the percussion test. This tooth presented: an attrition lesion on the occlusal surface of the tooth, a cervical buccal composite restoration of good clinical quality, a normal response to the cold test, an altered periodontal status evidenced by spicules of tartar, Grade 2 Mülheman tooth mobility and elevated probing depths (5 mm buccal and lingual and 6 mm mesial and distal), and a Periapical Index (PAI) of 2 (Figure 1A) (Ørstavik et al. 1986).

*Diagnostic Assessment:* The diagnostic hypothesis of the DEU was symptomatic apical periodontitis on Tooth#35 due to bruxism.

*Therapeutic intervention:* An occlusal reduction of the tooth was performed. The patient was referred to the endodontic and prosthetic department for follow‐up.

### 2.2 Second Visit: Endodontic and Prosthetic Department

*Clinical findings:* 4 weeks after the DEU visit, the patient reported severe spontaneous pain (VAS 8/10). Percussion test on tooth 35 was also painful cold test caused severe and prolonged pain on the tooth but elicited normal responses for the adjacent teeth (31, 32, 33, 34, and 37).

*Diagnostic Assessment:* Symptomatic irreversible pulpitis with periapical sensitization was the most probable diagnosis, but the cause was uncertain. It could be secondary to periodontal lesion, to composite restoration, or due to the occlusal load. Occlusal overload, supported by occlusal attrition, radiological findings, and mechanical sensitivity was the more probable.

*Therapeutic Intervention:* With due consideration of the medical condition of the patient, a root canal treatment was performed in one visit in accordance with guidelines (Goodell and Balson 2012; Moinzadeh et al. 2013), with prophylactic antibiotic administration (2 g of amoxicillin 1 h before the procedure). After local anesthesia and rubber dam isolation, the access cavity was prepared. Intense bleeding at the opening of the chamber supported the diagnosis of symptomatic irreversible pulpitis. Evidence of pulpal infection, including odor and purulence, was not detected. The working length was measured with an apex locator (Propex pixi, Dentsply Sirona, Ballaigues, Switzerland) and controlled during the procedure to avoid periapical overinstrumentation. Shaping was completed under irrigation with 3% sodium hypochlorite using reciprocating files (Wave one gold, Dentsply Sirona, Ballaigues, Switzerland). After a final rinse with 17% EDTA and 3% sodium hypochlorite solutions and manual activation, a hydraulic technique was used to fill the root canal system (Bioroot RCS, Septodont, St Maur des Fossés, France). The cavity access was then sealed with composite resin (Figure 1C,D), and localized periodontal dental scaling was performed at the end of the session.

### 2.3 Third Visit: Endodontic and Prosthetic Department

*Clinical findings:* The pain subsided for 3 days, but then intensified (VAS: 8/10), spreading to the entire left mandible the day after the ongoing chemotherapy (doxorubicin [myocet], cyclophosphamide [endoxan], paclitaxel [taxol]). All pulpal tests were within normal limits for the adjacent teeth (24, 23, 22, 21, and 18).

*Diagnostic Assessment:* Differential diagnosis was made between endodontic flare‐up and acute neuropathic chemotherapy‐induced pain. An endodontic flare‐up diagnosis was excluded because of (1) the pulpal state before root canal therapy (vital pulp and noninfected pulp), (2) absence of overinstrumentation and overfilling during the procedure, and (3) aseptic conditions of the endodontic treatment. Chemotherapy‐induced pain was then hypothesized.

*Therapeutic intervention*: Nefopam (20 mg/2 mL per os, twice a day) was prescribed.

### 2.4 Fourth Visit: Endodontic and Prosthetic Department

**Figure 2:**
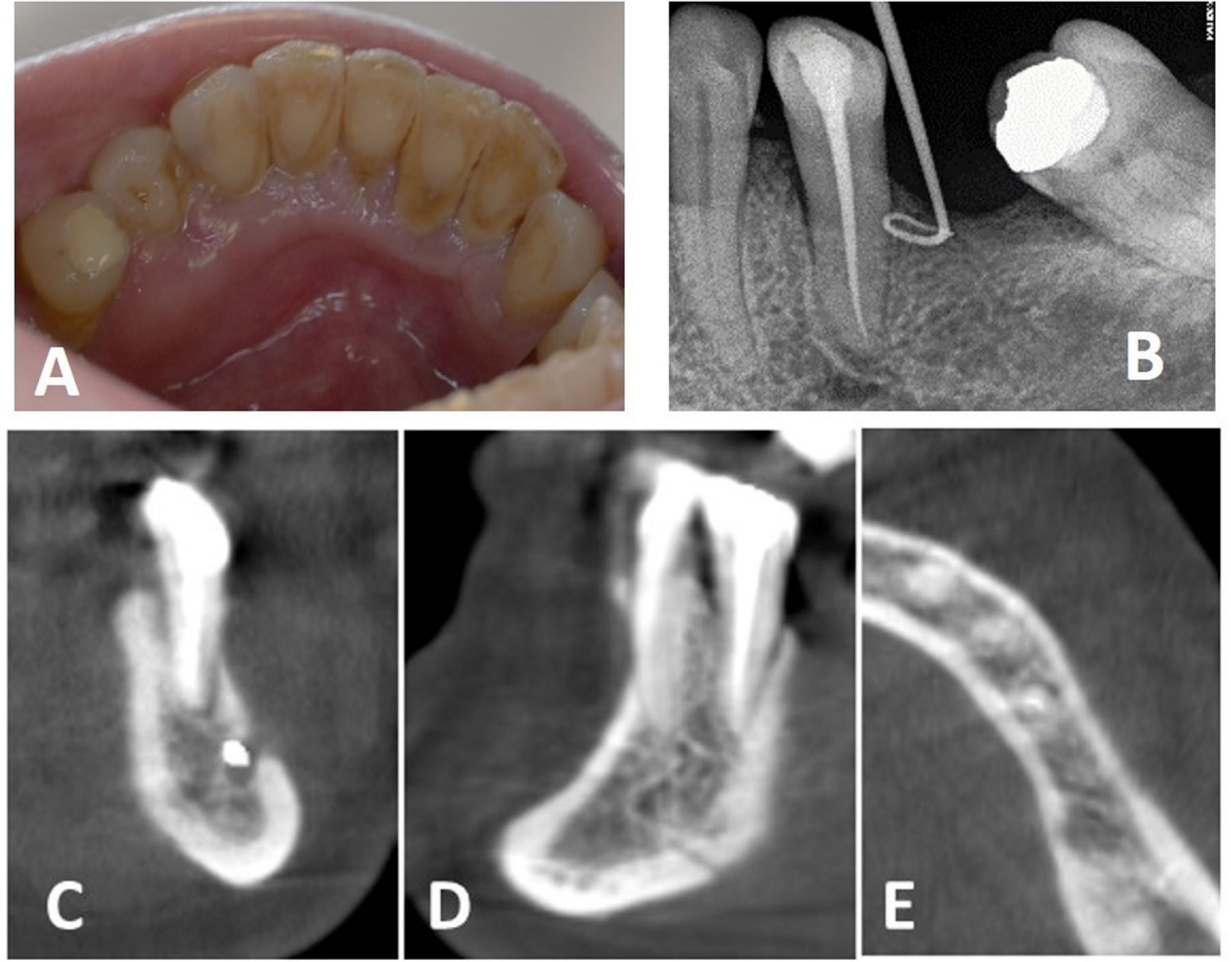
(A) Lingual tumefaction facing the lower left second premolar. Calculus is visible in the lingual part of the mandibular incisors. (B) X‐ray with a gutta‐percha cone inserted into the sinus tract identified the edentulous crest as the source of the pus. A PAI score of 2 was estimated for the lower left second premolar. (C–E) Frontal, sagittal and horizontal CBCT slices. A widening of the periodontal ligament is visible with no obvious changes in the structure and density of the alveolar bone.

*Clinical findings:* After 3 weeks, there was no change in pain intensity, but a sulcular purulent discharge and lingual tumefaction facing the tooth 35 were evidenced. The tooth 35 was mobile (Grade 4). An X‐ray with a gutta‐percha cone inserted into the sinus tract (located in the lingual mucosa of the lower left second premolar) evidenced an edentulous crest as the source of the abscess (Figure 2A,B).

*Diagnostic Assessment:* “Serpiginous abscess”, i.e., fistula in a nonclassical location regarding the origin of the lesion, as seen in anterior teeth after occlusal trauma was looked for but not found, confirming the periodontal origin of the abscess. Due to the patient's medical conditions (AR medication and cancer recurrence) and the aggravation of her symptomatology, an MRONJ was suspected.

*Therapeutic intervention*: A generalized dental scaling was performed during the clinical session and the patient was referred to the oral surgery department for their expertise in treating patients on AR drugs (Lescaille et al. 2014).

### 2.5 Fifth Visit: Oral Surgery Department

*Clinical findings:* A CBCT was prescribed to complete the clinical investigations (Figure 2C–E) according to guidelines (Campisi et al. 2020). A widening of the periodontal ligament was visible on the lower left second premolar, with no obvious changes in the structure and density of the alveolar bone.

*Diagnostic Assessment:* Despite the absence of clear radiological signs, but based on the clinical course, MRONJ was suspected.

**Figure 3:**
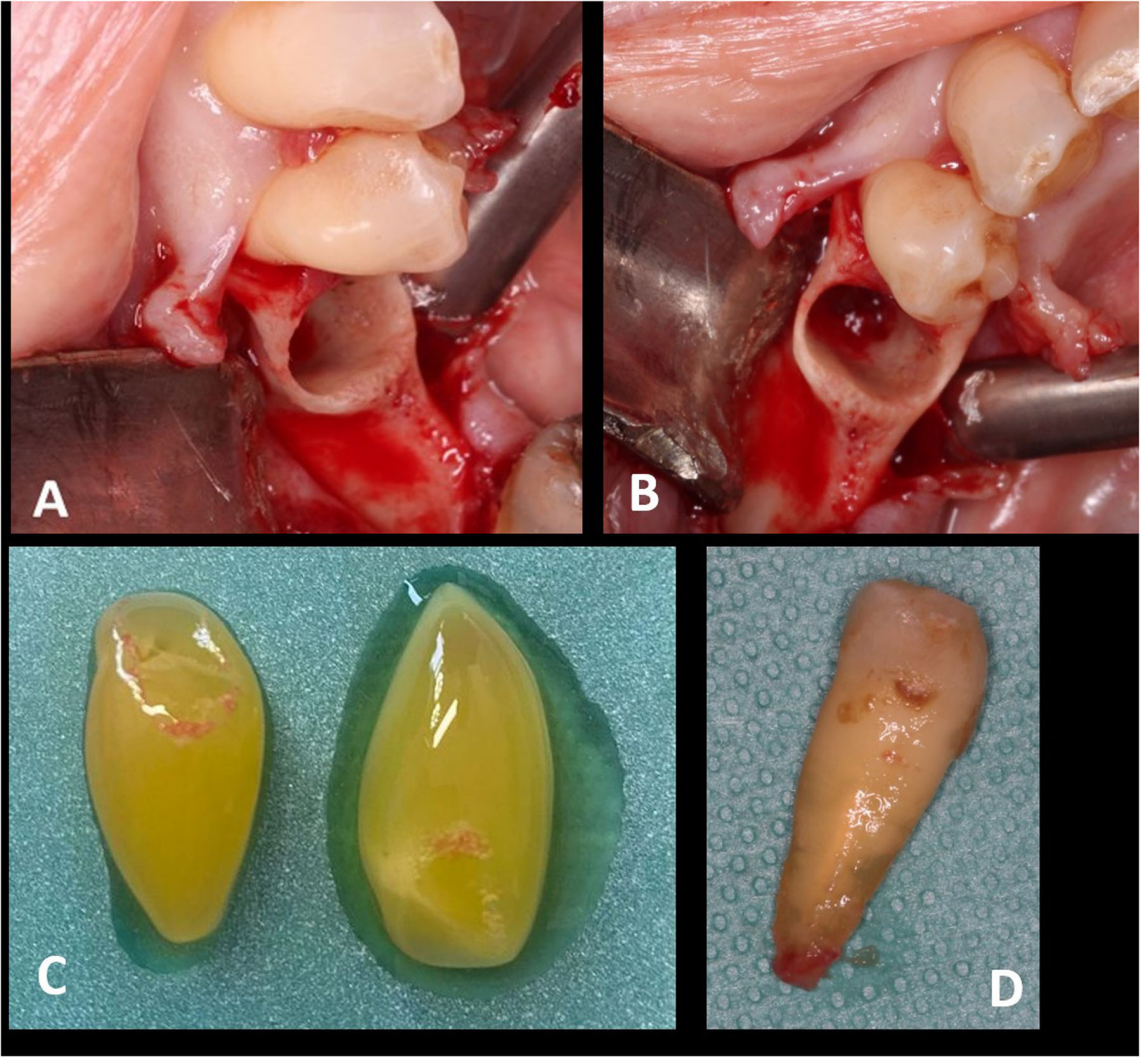
(A) Avascular bone after the lower left second premolar removal. (B) Bleeding induction after osteotomy of the socket. (C) Platelet‐rich fibrin (PRF). (D) The lower left second premolar.

*Therapeutic intervention:* Extraction of tooth 35 with amoxicillin (2 g) prophylaxis was decided to allow exploration of the bone. After tooth removal, necrotic bone was found in the socket of the lower left second premolar. A carefully executed localized osteotomy was performed to remove necrotic tissue and promote bleeding, followed by intra‐alveolar placement of platelet‐rich fibrin, and hermetic sutures (Figure 3). The minimally invasive osteotomy did not allow enough tissue to be collected for histologic analysis. Antibiotics (amoxicillin 2 g per day until the mucosal healing) and analgesics were prescribed (paracetamol 500 mg/codeine 30 mg four times a day for 4 days).

### 2.6 Sixth Visit: Oral Surgery Department

Because of the first COVID wave (March 2020), follow‐up of the patient occurred 4 months after the surgery.

*Clinical findings:* The patient reported spontaneous pain (VAS: 8/10) and recurrence of infection, which responded poorly to antibiotics (amoxicillin 2 g per day for 3 weeks). The tooth 34 no longer responded to cold and electric pulp tests, and its mobility was severely increased (Grade 4). Periodontal probing depth was within normal limits (probing depths were 5 mm buccal, lingual, mesial, and distal).

*Diagnostic Assessment:* The similarity of the clinical sequence was interpreted as an extension of the disease, which first occurred on the lower left second premolar and was taken as confirmation of the MRONJ diagnosis.

### 2.7 Seventh Visit: Oral Surgery Department

*Therapeutic intervention:* 5 months after the first surgery, extraction of tooth 34 with associated with a new osteotomy was performed. The socket was again filled with platelet concentrate and antibiotics (amoxicillin, 1 g twice a day, until mucosal healing) were prescribed.

**Figure 4:**
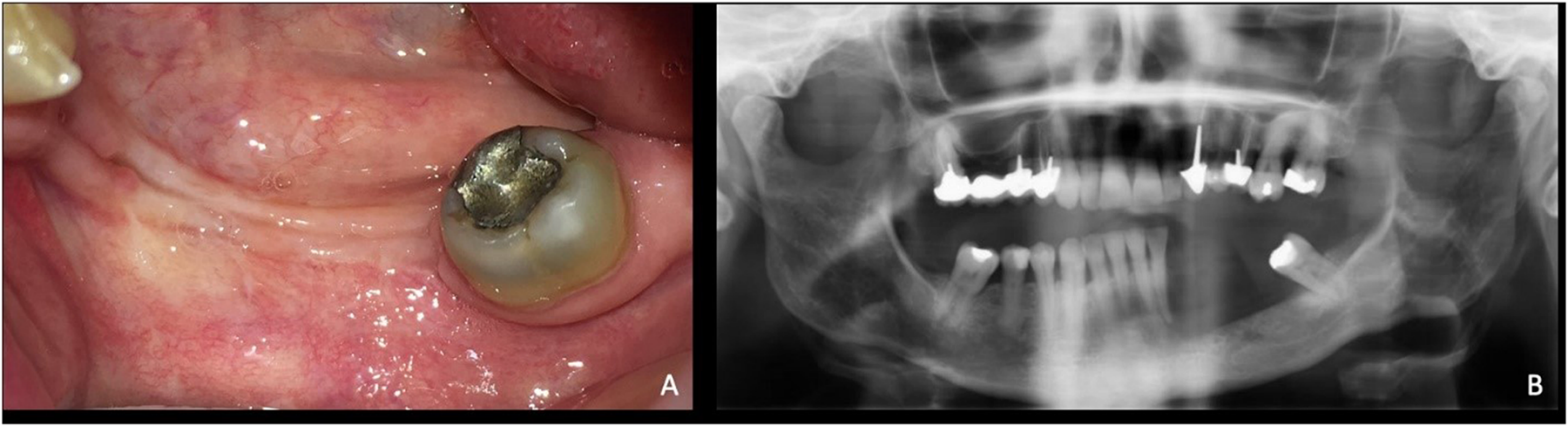
Control visit at 4 months after the second surgery. (A) Clinical view. (B) Radiological view.

*Eighth and ninth visits:* Postoperative controls at 3 weeks and 4 months showed complete healing of the operative site, with no clinical and radiological signs (Figure 4). As the lower left canine was vital and asymptomatic, with Grade 1 physiologic mobility despite a weak osseous support, the decision was made to keep it in place to avoid any additional bone trauma and to preserve prosthodontic possibilities.

Unfortunately, the patient died of COVID‐19 4 months after the last control visit.

## 3 Discussion

The clinical sequence of this case can be summarized as follows: undiagnosed MRONJ, modified local conditions, and impaired vascular bone supply. The occlusal trauma provoked apical periodontitis of the lower left second premolar. The combination of these events led to symptomatic irreversible pulpitis. The periodontal abscess evidenced by a fistula was a sequela to MRONJ. The lower left second premolar was removed to gain therapeutic access to periapical bone. Despite the curettage and placement of platelet concentrate, the disease continued and elicited symptomatic pulpitis of the lower left first premolar leading to its extraction. Antibiotic coverage appeared to prevent further complications. Tissue healing was observed before the patient's death.

An intriguing event is the occurrence of symptomatic pulpitis, first in the lower left second premolar. Usually, pulpitis is caused by pulpal exposure to bacteria, for example, after alteration of marginal sealing of a restoration. No such evidence was found. Pulpal infection can also be of periodontal origin, the severity of the pulpal reaction depending on the virulence of the periodontal pathogens and the capacity of pulpal repair (Bergenholtz and Lindhe 1978). However, pulpitis and pulp necrosis of periodontal origin are rare and usually associated with deep periodontal lesions (Simon et al. 2013), which was not the case here. Bacterial contamination could have been a consequence of reductions in pulpal pressure and blood flow (Heyeraas and Berggreen 1999), resulting from vascular impairment due to periodontal changes elicited by occlusal load (Cooke 1982).

A more plausible etiology, possibly acting in conjunction with occlusal overload, is MRONJ, which impairs vascularization of periodontal bone (Bedogni et al. 2008). If MRONJ can impair pulp vascularization to induce pulp necrosis “a retro” (Kiho et al. 2020), then it might also induce symptomatic pulp inflammation. The reasons for pain are not known, but deteriorating pulp generates algogenic mediators (Jain et al. 2013). Drilling, for example, can cause painful aseptic reversible inflammation due to neuropeptides released by axon reflex (Ngassapa 1996; Byers and Narhi 1999). Hypoxic conditions known to affect pulp homeostasis (Colombo et al. 2020) also promote pain in conditions such as sickle cell disease (Aich et al. 2019); several mechanisms such as sensitization of nociceptive TRPV1 receptors (Ristoiu et al. 2011) or algogenic action of Hypoxia Induced Factor 1‐alpha (HIF1a) can be hypothesized (Kanngiesser et al. 2014).

In our patient, multiple findings supported the pre‐existence of MRONJ at the emergency visit. For example, thickening of the lamina dura, widening of the ligament space, and periodontal bone loss, as observed in our patient, are common findings of early‐stage MRONJ (Bedogni et al. 2008; Moreno‐Rabié et al. 2020). Moreover, the progressive aggravation of the symptomatology despite the therapeutics, the symptomatic pulpitis despite the occlusal reduction, the bone loss despite the periodontal scaling and the occurrence of a periodontal abscess for the lower left second premolar, the repetition of the clinical sequence in the lower left first premolar (i.e., pulpal necrosis despite a vital test a few weeks prior), the rapidity of the loss of osseous bone support, the medical context of the patient, and the appearance of the bone during surgery, all confirm *a posteriori* the diagnosis of MRONJ. Onset of MRONJ can be spontaneous in high‐risk patients (Mavrokokki et al. 2007), but might have been induced in our patient by periodontal pathogens (Tsao et al. 2013) or bruxism (Mine et al. 2022) or by a combination of the two.

Another intriguing clinical observation was the exacerbation of the symptomatology and the presence of purulent discharge. The sinus tract was not of endodontic origin, as evidenced by the gutta‐percha cone. The bacterial contamination might be attributed to periodontal pathogens (periodontal disease) or consecutive to our therapeutics, although the latter is very unlikely given the specific attention paid to avoid trauma and bacterial contamination during the endodontic procedure. *Actinobacillus actinomycetemcomitans* and *Porphyromonas gingivalis* present in periodontitis favor bone erosion in animal models with lipopolysaccharide acting as a virulence factor (Nishida et al. 2001). This may explain the extension of MRONJ. The inability of osteoclasts to remove bacteria‐infected bone in MRONJ lesions leads to osteonecrosis, its extension, pus formation, and fistula drainage (Williams et al. 2014).

This case report adds to that of Kiho et al. (2020), both sounding a warning to endodontists of the possible endodontic consequences of MRONJ. The development of osteolysis and impaired vascularization near a tooth may lead to pulp inflammation and necrosis. It is therefore crucial to regularly monitor the pulpal state of the teeth of patients treated with AR drugs, especially in high‐risk patients (Table 1C). The presence of periapical radiolucency despite a vital pulp associated with a thickened lamina dura, as observed on the lower left second premolar of our patient, should be considered as a warning sign in patients treated with AR drugs.

When MRONJ is diagnosed, endodontics is recommended to decrease the risk of infectious spreading (Hadaya et al. 2019). Moreover, the rapid spread of MRONJ encourages early follow‐up. In the case of high‐risk patients (Campisi et al. 2020), the recommended 6‐month follow‐up should be modified to include follow‐ups at 1 month, 6 months, and 1 year after an endodontic procedure.

As a conclusion, AR drugs can impair oral health. As reported here, MRONJ can lead to pulpal inflammation and necrosis. Diagnosis can be difficult (especially at Stage 0) without clear radiographic signs, and endodontists should be aware of the clinical consequences of MRONJ.

## Author Contributions

Data collection and analysis: Vanessa Baaroun, Samantha Elbhar, and Ines Guessoum. Drafting of the manuscript: Vanessa Baaroun, Samantha Elbhar, Carole Rémond, and Marjorie Zanini. Critical review: Juliette Rochefort, Geraldine Lescaille, and Yves Boucher. All authors have contributed significantly. All authors read the final article and approved the version submitted for publication.

## Consent

The patient gave informed written consent.

## Conflicts of Interest

The authors declare no conflicts of interest.

## Data Availability

The authors have nothing to report.
